# Use of Whole Genome Sequencing to Determine the Microevolution of *Mycobacterium tuberculosis* during an Outbreak

**DOI:** 10.1371/journal.pone.0058235

**Published:** 2013-03-05

**Authors:** Midori Kato-Maeda, Christine Ho, Ben Passarelli, Niaz Banaei, Jennifer Grinsdale, Laura Flores, Jillian Anderson, Megan Murray, Graham Rose, L. Masae Kawamura, Nader Pourmand, Muhammad A. Tariq, Sebastien Gagneux, Philip C. Hopewell

**Affiliations:** 1 Curry International Tuberculosis Center, Division of Pulmonary and Critical Care Medicine, University of California San Francisco, San Francisco, California, United States of America; 2 Tuberculosis Control Section, San Francisco Department of Public Health, San Francisco, California, United States of America; 3 Centers for Disease Control (CDC), Division of TB Elimination, Field Services and Evaluation Branch (CDC/DTBE/FSEB), Atlanta, Georgia, United States of America; 4 Department of Bioengineering, Stanford University, Palo Alto, California, United States of America; 5 Department of Pathology, Stanford University, Palo Alto, California, United States of America; 6 Department of Epidemiology, Harvard School of Public Health, Boston, Massachusetts, United States of America; 7 MRC National Institute for Medical Research, London, United Kingdom; 8 Department of Biomolecular Engineering, University of California Santa Cruz, Santa Cruz, California, United States of America; 9 Swiss Tropical and Public Health Institute, Basel, Switzerland; 10 University of Basel, Basel, Switzerland; University of California, United States of America

## Abstract

**Rationale:**

Current tools available to study the molecular epidemiology of tuberculosis do not provide information about the directionality and sequence of transmission for tuberculosis cases occurring over a short period of time, such as during an outbreak. Recently, whole genome sequencing has been used to study molecular epidemiology of *Mycobacterium tuberculosis* over short time periods.

**Objective:**

To describe the microevolution of *M. tuberculosis* during an outbreak caused by one drug-susceptible strain.

**Method and Measurements:**

We included 9 patients with tuberculosis diagnosed during a period of 22 months, from a population-based study of the molecular epidemiology in San Francisco. Whole genome sequencing was performed using Illumina’s sequencing by synthesis technology. A custom program written in Python was used to determine single nucleotide polymorphisms which were confirmed by PCR product Sanger sequencing.

**Main results:**

We obtained an average of 95.7% (94.1–96.9%) coverage for each isolate and an average fold read depth of 73 (1 to 250). We found 7 single nucleotide polymorphisms among the 9 isolates. The single nucleotide polymorphisms data confirmed all except one known epidemiological link. The outbreak strain resulted in 5 bacterial variants originating from the index case A1 with 0–2 mutations per transmission event that resulted in a secondary case.

**Conclusions:**

Whole genome sequencing analysis from a recent outbreak of tuberculosis enabled us to identify microevolutionary events observable during transmission, to determine 0–2 single nucleotide polymorphisms per transmission event that resulted in a secondary case, and to identify new epidemiologic links in the chain of transmission.

## Introduction

Genotyping of *Mycobacterium tuberculosis* has been used to determine whether two or more isolates of *M. tuberculosis* are of the same strain. The markers used for genotyping include insertion sequence (IS) *6110* [1], direct repeat region [2], mycobacterial interspersed repetitive units-variable number tandem repeats (MIRU-VNTR) [3], and the polymorphic GC-rich sequence (PGRS ) [4]. If two patients have tuberculosis (TB) due to the same strain, these patients are considered to be part of the same chain of transmission [5]. If a patient isolate has a unique genotype (a genotype not isolated from other patients within the population), it is assumed that the disease is the result of progression to active disease from latent infection. In population-based studies over longer time periods (e.g., more than 3 years) *M. tuberculosis* genotype can be used to track a specific strain in the population. However, when TB cases occur within a short time window, such as during an outbreak, the directionality and sequence of transmission cannot be established using the current genotyping tools.

Recently, whole genome sequencing (WGS) has been used to describe the molecular epidemiology of several pathogens including *M. tuberculosis* [6]–[11]. This work has shown that single nucleotide polymorphisms (SNPs) identified by WGS were useful in identifying transmission events involving *M. tuberculosis* isolates that were genetically indistinguishable by IS*6110* Restriction Fragment Length Polymorphism (RFLP) or MIRU-VNTR typing [8], [10], [11]. Identification and analysis of SNPs enabled identification of multiple patients as sources of *M. tuberculosis* transmission. However, in one study, not all the *M. tuberculosis* isolates involved in the chains of transmission were analyzed [8], and in another, the SNPs identified by WGS analysis were not confirmed by other methodologies such as PCR Sanger method [10].

The objectives of this study were to use WGS to 1) describe the microevolution of all *M. tuberculosis* isolates identified during a well-characterized outbreak of drug-susceptible TB; 2) establish the sequence and directionality of each of the transmission events using WGS and epidemiologic data; 3) estimate the number of mutations per transmission event that resulted in a secondary case; and 4) estimate the possible functional consequences of non-synonymous SNPs (nsSNPs).

## Materials and Methods

### Study Population

The protocols and procedures for the protection of human participants were approved by the University of California, San Francisco (UCSF) Human Research Protection Program which is equipped to provide ethical review and consultation. Consent was not obtained as we used information routinely collected as part of standard of care. We analyzed 9 *M. tuberculosis* isolates from 8 culture positive TB cases associated with an outbreak that was identified in an ongoing population-based molecular epidemiology study of TB in San Francisco, CA [12] and one isolate from a patient in Santa Clara, CA with the same genotyping identified in the TB GIMS (TB Genotyping Information Management System [13]. The *M. tuberculosis* isolates were isolated from sputum samples collected by TB Clinic staff as part of the diagnostic evaluation for TB and were sent to the San Francisco or Santa Clara County Public Health Laboratory per standard protocol. All initial isolates of *M. tuberculosis* from San Francisco are sent to our laboratory for genotyping where the strains are analyzed anonymously. The isolate from Santa Clara was specifically requested for further analysis. Genotyping is a standard microbiological evaluation in the United States and more refined and accurate genotyping methods are regularly being evaluated in comparison with the standard methods.

### Contact Investigation

The TB Control Section of the San Francisco performs contact investigation when a patient is diagnosed with culture-positive pulmonary TB. This procedure involves a patient interview and home visit to elicit contacts, as well as a patient re-interview. Contacts living in San Francisco that have been identified by the index case are then invited to be evaluated for active and latent TB through symptom screening and Mantoux tuberculin skin testing (TST) or interferon gamma release assay (IGRA). Medical evaluation and chest x-ray is performed if the TST, IGRA or symptom screening is positive. Routine procedures were followed for this contact investigation.

### Whole Genome Sequencing and SNP Detection and Confirmation

DNA from *M. tuberculosis* isolates from the 9 persons with culture positive TB was extracted using standardized procedures from Lowenstein Jensen media (first subculture from the original liquid culture) in all cases except isolate E, in which DNA was extracted from the second subculture [14]. One microgram of DNA from *M. tuberculosis* was used to prepare libraries for Next-Generation Sequencing (NGS) according to the manufacturer’s instruction using Illumina Genomic Sample pre kits (Illumina, Inc, San Diego, CA) or New England Biolabs library preparation kits (Ipswich, MA 01938-2723). The *M. tuberculosis* DNA libraries were sequenced on Genome Analyzer (Illumina, Inc, San Diego, CA) for WSG according to the manufacturer’s specifications. Single-end Illumina reads were independently aligned to *M. tuberculosis* reference strain H37Rv using BWA (Burrows-Wheeler aligner, http://bio-bwa.sourceforge.net/). Regions around loci with candidate insertions and deletions were realigned using GATK (Genome Analysis Toolkit, version 1.0.5974, http://www.broadinstitute.org/gsa/wiki/index.php/The_Genome_Analysis_Toolkit). Alignment output for each sample was indexed, sorted and merged into a single alignment file with SAM tools (version 0.1.17). Base calls (sequence deduced by the WGS signal) for each locus were called using the Unified Genotyper from GATK http://www.1000genomes.org/wiki/Analysis/Variant%20Call%20Format/vcf-variant-call-format-version-40). Each record includes a consensus base call, a quality metric and listing of the base pileup. A custom program written in Python performed a pairwise comparison of sample pileup outputs, inferring similarities and differences from the GATK output. Loci were annotated using the H37Rv annotations, accession number NC_000962. For coding sequences, the resulting amino acid translations were reported.

### SNP Detection and Confirmation

SNPs observed in large paralog gene families such as PE, PE_PGRS, PPE genes (gene families with highly conserved and repetitive sequences) and mobile elements were excluded due to potential mapping errors. Twenty five putative SNPs, in which 85% or more of the reads were consistent with one base call and had at least 12 reads depth, were analyzed with PCR Sanger method. DNA sequence data is publicly available in GenBank. Seven were confirmed as true SNPs. 1microM of each forward and reverse primers (Table S1) and 100–500 ng of DNA were added to FailSafe PCR 2X PreMix L (Epicentre, Madison, WI) for SNPs 1 and 6 and PreMix B for SNPs 4, 5, and 8. For SNPs 3 and 7, 8 uM of both forward and reverse primers and 300–500 ng of DNA were added to 1X of GoTaq Green solution (Promega Corporation, Madison, WI). PCR was performed with the following parameters: denaturation of 95°C for 3 min, 35 cycles of amplification (95°C for 60 sec, 55°C for 60 sec and 72°C for 60 sec (50 sec for SNP8), and elongation at 72°C for 5 min. PCR products were sequenced at the UCSF Genomic Core Facility using ABI Big-Dye v3.1 dye terminator sequencing chemistry (Applied Biosystems, Carlsbad, CA) and the ABI PRISM 3730xl capillary DNA analyzer. Sequence data were analyzed using ApE v1.17 and basic local search alignment was performed using NCBI’s genomic BLAST (http://blast.ncbi.nlm.nih.gov/Blast.cgi).

### Analysis

#### Phylogenomic inference

In order to determine evolutionary relationships of the isolates, we used the concatenate approach, in which all SNPs were concatenated to create a genotype for each isolate.

#### SNP-Based transmission link

We used the transmission tree approach in which the ancestors and their descendants are both present in the sample analyzed [15]. We used the maximum parsimony genealogy according to the number of mutations between pairs of isolates.

#### Predicting the functional effect of mutations

We used the Sorting Tolerant From Intolerant (SIFT) algorithm [16] to predict the mutations most likely to affect protein function. SIFT searches for homologs in other bacteria of the gene of interest and 1) scores the conservation of the positions where mutations are found, and 2) weights this score by the nature of the amino acid change. These measures are then incorporated into a proxy for impact of a specific mutation on protein function. Mutated positions with normalized probabilities less than 0.05 are predicted to have an impact on the protein and those greater than or equal to 0.05 are predicted to not impact the protein [16]. Homologs from two databases were used to form the predictions. One contained the 13 available complete mycobacterial genomes that are not in the *M. tuberculosis* complex. The second database used the complete genomes from the current SWISS-PROT & TrEMBL sequence database. Predictions made by the SIFT algorithm can only be reliable if the homologs used to generate the sequence alignment, and the following substitution matrix, are diverse enough to reflect the tolerance of the sequence to amino acid variation. In other words, SIFT could predict a non-synonymous SNP (nsSNP) having a functional impact on protein function just because the sequences used are very similar, leading to a high number of false-positives. To be confident in the predictions, we used the SIFT conservation metric (http://sift.bii.a-star.edu.sg/www/SIFT_help.html). This provides a measure of sequence diversity within an alignment and demonstrates confidence in the SIFT predictions.

## Results

### *M. tuberculosis* Outbreak Strain

We identified 9 Hispanic young male adults (average age of 27 years, range 18 to 34 years), all with negative HIV tests, with *M. tuberculosis* isolates that had matching genotypes. All patients were foreign-born and eight reported living in San Francisco an average of 32 months with a range of 6–77 months, and one patient reported living in Santa Clara for 16 months. All isolates from this outbreak were susceptible to isoniazid, rifampin, ethambutol, pyrazinamide and streptomycin and were genetically indistinguishable based on having 8 identical IS*6110-* hybridizing bands, a spoligotype with an octal code of 777777774020731, and a 12-locus MIRU-VNTR pattern of 225223153424. This genotype (based on spoligo and MIRU typing) has only been documented in the cases reported in this paper, based on the review performed on November 28, 2012 of the genotyping databases of San Francisco, the TB GIMS (TB Genotyping Information Management System at CDC [13] and the MIRU-VNTR database (http://www.miru-vntrplus.org/MIRU/batchIdentification.faces) which contains MIRU information from isolates from different parts of the world.

**Figure 1:**
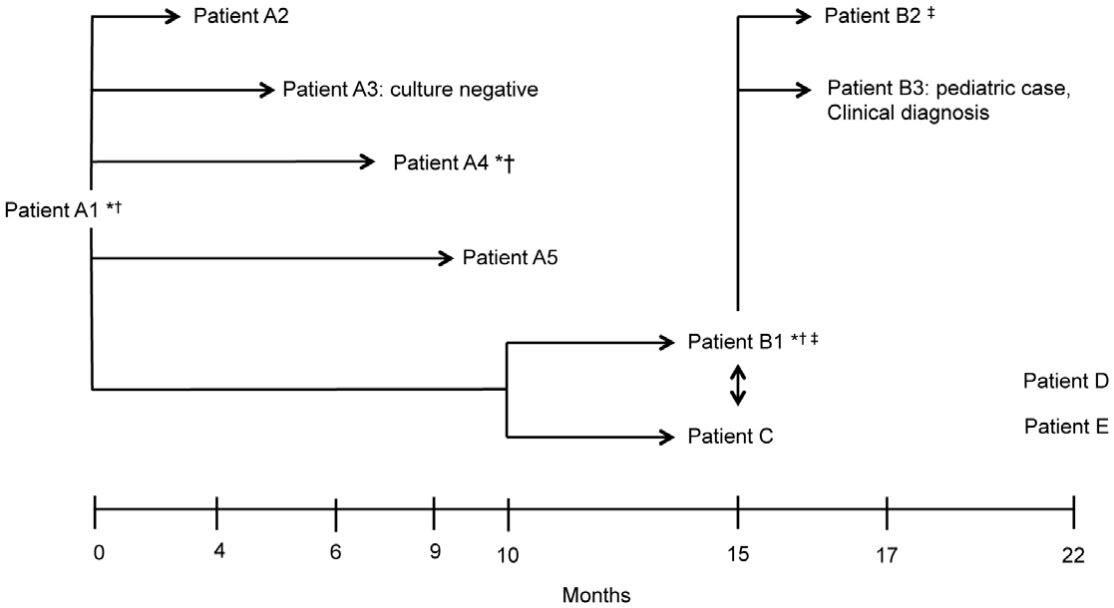
Timeline (months) and characteristics of the patients involved in the outbreak. The epidemiological links are shown with arrows. *Chest radiograph showing cavitation. †Positive sputum smear examination. ‡Laryngeal tuberculosis.

The outbreak strain was first identified in patient A1 (Figure 1). The patient presented with one month of productive cough, chills, night sweats, and weight loss of 5 kg and was found to have pulmonary TB with multiple left upper lobe cavities. During the contact investigation for A1, 25 of 30 (83%) household contacts living in crowded conditions had either a positive IGRA test (QuantiFERON-TB Gold In-Tube, Cellestis, Valencia, CA, USA (QFT)) or a positive TST. Two contacts had negative TST results and three had a history of a prior positive result and were not re-tested. Two of the contacts with a positive test were found to have TB disease at the time of the initial evaluation (A2 and A3, Figure 1). During the contact investigations for A2 and A3, patients A4 and A5, (Figure 1) were identified as former residents of A1’s household but were not living in the house at the time the residents were screened for TB, and, thus, were not included in the initial investigation.

Fourteen months after A1 was found, patient B1 presented to the health department with one year of cough, fever, hoarseness, and weight loss and was found to have laryngeal and pulmonary TB with multiple cavitary lesions. During the contact investigation for patient B1, 21 of 29 (76%) exposed persons had a positive QFT or TST, and five had a negative QFT (4 had a follow-up negative test and one did not receive the second test). Additionally, two contacts had a history of prior positive TST results and were not re-tested, and one other contact was not tested. Among the contacts of patient B1, two cases of TB disease were found (B2 and B3, Figure 1). Patient B3 was a pediatric case; no clinical samples were collected and the clinical diagnosis of TB was based on a chest radiograph and clinical symptoms which improved with treatment. Further questioning of patients B1 and A1 determined these patients knew each other, but patient B1 was not named as a contact by A1, and was, therefore, not screened during the contact investigation for A1.

Patient C was also found to have TB disease, diagnosed 14 months after A1, but was not identified through contact investigations for patients A1 or B1. However, during the contact investigation of patient C, it was found that patient C knew patients A1 and B1. Twenty-two months after the diagnosis of A1, patients D and E were found to have TB disease. Patient D presented for medical evaluation with 2 weeks of cough and fever and experienced minor weight loss over 1 week; the contact investigation did not find any epidemiological links with the previous cases. Patient E reported experiencing night sweats for four months prior to diagnosis. This patient was diagnosed with extra-pulmonary TB and had a positive culture of fluid aspirated from the cervical lymph node. Contact investigation was not performed because the disease was only extra-pulmonary. All patients lived in San Francisco except patient E, who lived in Santa Clara (72.5 km from San Francisco). The focus of this study is on the nine culture positive cases of the 11-person outbreak.

*WGS results and confirmed SNPs*. We obtained between 4 and 7 million reads of 36 bp each per *M. tuberculosis* isolate sequenced. The coverage of the WGS data for each isolate (based on the genome size of the reference laboratory strain H37Rv) was between 94.1% and 96.9%, with an average of 95.7%. The read depth (number of times a particular locus was sequenced) varied from 1 to 250, with an average of 73 times. The WGS of isolate A1 (the first case) was used as a reference to which the other eight sequences were compared. The sequences of the nine isolates were identical except for seven SNPs (Table 1 and Table S2). Based on the methodology described, we did not identify any insertions or deletions among the isolates involved in the outbreak. Two mutations were synonymous SNPs (sSNPs) and five were nsSNPs. The nsSNPs were unique to our strains (not lineage-specific) based on a search performed in two sets of lineage-specific nsSNPs from genome sequences of a representative global collection of *M. tuberculosis* strains published previously [17], [18].

**Table 1:** Characteristics of the SNPs observed among the nine *M. tuberculosis* isolates.

SNP	Position (in H37Rv)	Gene name	Function	Codon in A1/codon in mutated isolate	Mutation	Gene Information	SIFT Score†	SIFT conservation†
1	172708	Rv0146	Possible methyltransferase.	TGG/TGT	W 166 C	Non-essential [27] *. Gene found to be partially deleted [28].	0.00/0.00	2.83/2.80
3	360546	Rv0296c	Sulfase	GCC/TCC	A 204 S	Non-essential [27] *	0.74/0.43	3.17/2.82
4	2956613	Rv2629	Conserved hypothetical	CGT/TGT	R 283 C	Induced by hypoxia [22], [23]	0.00/0.00	3.42/3.00
5	2968379	Rv2643 *arsc*	Probable arsenic-transport integral membrane protein	GAG/GAC	E 490 D	Putative heavy metal resistance membrane protein [29]	0.18/0.18	4.32/4.32
6	3492039	Rv3126c	Hypothetical protein	CGC/CGT	Synonymous R 68 R	Non-essential [27] ^1^.Induced by hypoxia [23]		
7	4124773	Rv3683	Hypothetical protein	GAC/GAT	Synonymous D 359 D	Non-essential [27] *		
8	3188412	Rv2877c	integral membrane protein	GGG/AGG	G 154 R	Non-essential [27] *Part of an operon that responds to a sigma factor regulating genes involved in the polyketide-lipid synthesis and in post-translational protein modification.	0.06/0.07	3.01/3.06

* By Himar1-based transposon mutagenesis in H37Rv strain culture in vitro [27].

† The first value is based on the SIFT prediction using the 13 non-*M. tuberculosis* complex genomes and the second value is based on the SWISS-PROT & TrEMBL sequence database.

The affected genes and their characteristics are shown in Table 1. SIFT analyses predicted that two of the five nsSNPs would have an impact on protein function (SNP1: Rv0146: W166C; SNP4: Rv2629: R283C). The SIFT analysis on SNP8 (Rv2877c G154R) predicted that the SNP is tolerated (score of 0.06 using the non-*M. tuberculosis* complex database and 0.07 using the SWISS-PROT & TrEMBL sequence database).

We further reviewed the WGS data to determine the presence of heterozygous base calls (presence of more than one base) in the loci corresponding to the seven SNPs, which had an average and median number of reads per loci of 133 (range 88 to 240). We found that the isolate from patient D (which did not show any SNPs based on the PCR sequence) had heterozygous base calls in the locus corresponding to SNP5; 38% of the reads corresponded to the mutation and the rest to the reference strain (pile depth of 75).

### Confirmation of the Contact Investigation-Based Links

**Figure 2:**
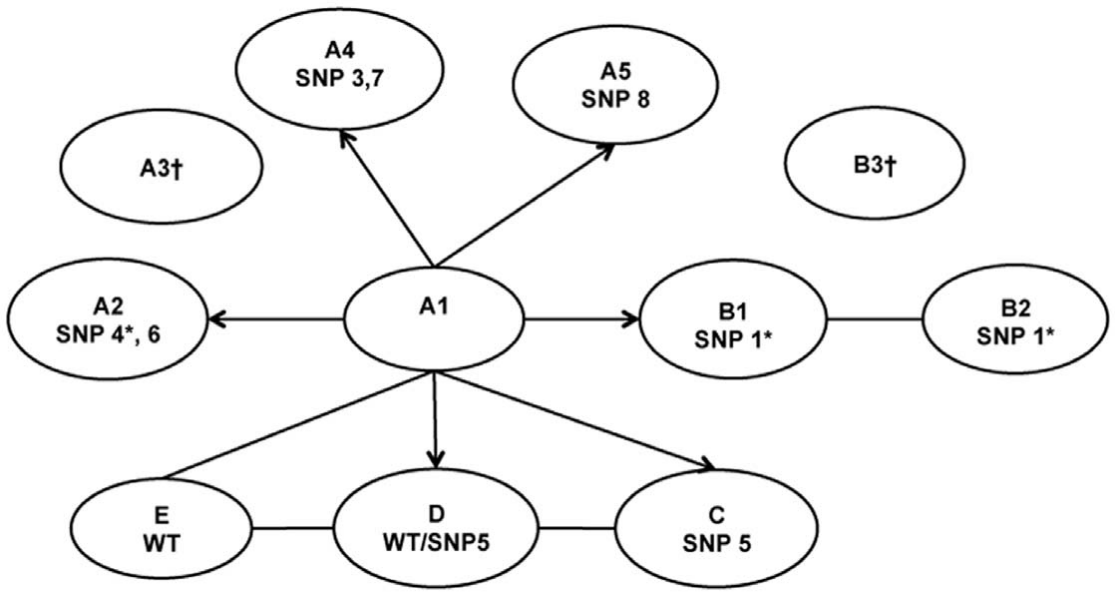
SNP-based network. The letters inside the circles correspond to the patients; SNP correspond to the number of the SNP observed in the *M. tuberculosis* isolate and described in Table 1; lines represent links based on the SNPs and arrows show the sequential SNP acquisition. WT refers to the genotype of A1. *Mutations predicted to affect the protein according to SIFT. †Culture negative or not performed.

Analysis of the SNPs confirmed that patient A1, who was the index case based on the epidemiological data, was the first case in the chain of transmission. The WGS data confirmed the epidemiological links identified through contact investigation methods, except for the link between patient B1 and C (Figure 2). Patient A1 was the source of infection for patients A2, A4, A5, B1 C and E and probably D. WGS showed that patient C was infected by patient A1 or D (Figure 2) and not by patient B. Based on the SNPs, patient D had a mixed population compatible with the *M. tuberculosis* isolate from patient A1 and patient C. Based on the WGS, the isolates from patient A1 and D and E were the same, but no epidemiologic links were reported. However, given that patients D and E have similar demographic profiles and time of diagnoses, we cannot rule out the possibility that these patients may have had contact with one of the other tuberculosis cases at some point in time.

The WGS data showed that during this outbreak, the isolate from patient A1 was the source of at least 5 different *M. tuberculosis* isolate variants, with 0–2 mutations per transmission event that resulted in a secondary case.

## Discussion

WGS analysis of all *M. tuberculosis* isolates from a recent outbreak of TB enabled us to 1) identify microevolutionary events within a chain of transmission, 2) confirm the epidemiologic links and the directionality of the chain of transmission, and 3) determine the number of SNPs per transmission event during this outbreak.

The median time of diagnosis between patient A1 and the 5 patients with confirmed transmission based on the WGS data was 9 months (range 4 to 15 months), and the median time of diagnosis between B1 and B2 was one month. Both patient A1 and B1 had multiple cavities, an indicator of high bacterial burden and a factor associated with a patient’s increased ability to transmit infection [19]. Although none of these patients were infected with HIV, a known factor for rapid progression to disease, a recent analysis of TB outbreaks in the US found that most patients did not have established medical risk factors (although not all patients were tested for HIV), but they did have social factors for TB [20]. In our study population, all patients were recent immigrants to US with an average stay of 30 months in the country, and may have had multiple factors that increased the risk of developing active TB [21]. While we cannot rule out the possibility that patients were infected by the same bacteria in a previous outbreak and that they all reactivated over a 22-month period, the likelihood of 9 patients developing active TB during a short period by the same unique latent strain is unlikely. The MIRU genotype of this *M. tuberculosis* strain was not found in the genotyping databases from San Francisco, California, USA, the CDC TB GIMS (TB Genotyping Information Management System) and the international MIRU-VNTR website (November 28, 2012), which suggest that this may not be a prevalent *M. tuberculosis* strain.

The sequences of all patients with the outbreak strain were identical except for seven SNPs that were confirmed by Sanger sequencing. These polymorphisms were useful in identifying genetic variations in a well described-outbreak. These data, together with detailed epidemiologic information, enabled us to study the microevolutionary events during transmission in detail. The genomic and epidemiologic data indicated that one patient (A1) transmitted *M. tuberculosis* to multiple contacts, and only one of these contacts became a source of secondary cases (B1). The use of WGS to identify a single additional source of infection is an important finding. By relying solely on the clinical and epidemiologic data, ascertainment of the directionality and sequence of transmission was difficult since the cases were all diagnosed within a short period of time.

As indicated by WGS, the *M. tuberculosis* isolate from patient A1 resulted in five different variants of *M. tuberculosis.* We do not know if these mutations occurred randomly and then expanded or were product of natural selection. We cannot rule out the possibility that some of the mutations may have impacted the pathogen’s transmissibility and ability to cause active disease. The *M. tuberculosis* from patient A2 (Figure 2) had an nsSNP in Rv2629, a gene induced during hypoxia [22], [23]. The isolate from B1 had a nsSNP in Rv0146, a gene involved in the innate responses of primary macrophages [24]. The SIFT scores of these genes suggested that these mutations may have an impact on the protein function. Further research is required to determine what effect, if any, mutations occurring during transmission events have on the pathogen, and if these mutations are the result of chance or the result of the transmission event acting as a selective force on the bacterial population.

The number of SNPs we found was much lower than the 204 SNPs reported by Gardy et al. [10] who performed WGS of 32 *M. tuberculosis* isolates from an outbreak using the same sequencing platform that we used. The large discrepancy between the numbers of SNPs found by Gardy et al. [10] and our study is probably due to the criteria used to define a SNP. Based on the reported methodology, Gardy et al. [10] did not confirm the data obtained by WGS. They also included 76 SNPs located in paralog gene families, some of which may be false SNPs due to mapping errors. SNPs detected by WGS in these genes were not further analyzed in our study and we only included WGS detected SNPs in which more than 85% of the base calls were homozygotes and that were subsequently confirmed by PCR sequencing. The number of mutations we found is similar to what was reported by Schürch et al. who performed WGS analysis on three isolates (from years 1992, 2004 and 2006) of *M. tuberculosis* that had the same IS*6110-*RFLP genotyping pattern [8], [9]. These isolates were part of a prevalent cluster in the community that was composed of 104 cases identified during 1992–2008, and the authors found eight SNPs among the three sequenced isolates. These SNPs were subsequently investigated in all 104 isolates of the cluster, resulting in the identification of secondary and tertiary source patients. It is interesting that the number of SNPs from a strain prevalent in one population for 14 years is similar to the number of SNPs from a non-prevalent strain causing an outbreak over a 22-month period. This may be partly explained by the fact that not all the isolates were sequenced by Schürch et al. Recently, Walker sequenced 114 paired isolates from individuals and household members with tuberculosis, and in 96% of the cases; the whole genome differed by five or fewer SNPs, which is compatible with our finding [11].

WGS verified the epidemiologic links established through contact investigation and confirmed index case A1 as the source case of the outbreak. Additionally, WGS was able to show that it was patient A1, not patient B1, that transmitted the TB organism to patient C. Additionally, patient D (who was symptomatic 2 weeks before diagnosis and almost 22 months and 7 months after patients A1 and C were diagnosed, respectively) had a mixed population of *M. tuberculosis* compatible with *M. tuberculosis* isolates from patients A1 and C. It is possible that patient A1 may have had both the wild-type *M. tuberculosis* population as well as a small population of the SNP5 mutant, which was not cultured or detected by WGS, who then transmitted these isolates to both patient C and patient D. The likelihood that patient D is the link between A1 and C is low, as the timeline is not compatible with this link and the contact investigation did not reveal any epidemiologic links. Similarly, we did not find epidemiological links to patient E who became symptomatic 4 months before diagnosis and was diagnosed 22 months after patient A1.

WGS was able to provide a link between cases that did not otherwise have an epidemiologic link. In patients with an identical strain, WGS indicated the sequence and direction of transmission, revealed the mutations acquired between transmission events, and gave an indication as to whether those mutations would affect the fitness of an organism or not.

Although WGS, combined with epidemiologic data, enables us to determine the most likely sources of infection, it is not possible to determine if the mutations occurred during the time *M. tuberculosis* was replicating in the index case, or if the mutations occurred in the secondary cases after transmission. Therefore, it is not possible to estimate mutation rate per number of generations of *M. tuberculosis* or per unit of time [25].

WGS has the potential to become an important molecular epidemiologic tool as it provides information about the microevolution of a strain during transmission as well as the source(s) of infection and the sequence of transmission events. This will be important as it can distinguish patients who are part of a recent chain of transmission from those with disease resulting from progression of remote infection. Moreover, WGS can be used in areas where the isolates causing TB are genetically similar (prevalent *M. tuberculosis* stains) and where current tools cannot distinguish between recent transmission and prevalent strains. Applying WGS to large population-based studies is still limited by the available laboratory and analytic tools. More importantly, the programmatic utility of WGS needs to be defined.

Our study has limitations. The WGS was performed on DNA extracted from *M. tuberculosis* sub-cultured from the original liquid media. It is possible that the culture process may have caused a bottleneck and may not reflect the true genetic make-up of the bacteria causing the disease in the patient. Direct sequencing of *M. tuberculosis* from clinical specimens would eliminate such concerns. However, the SNP-based network correlated with most of the clinical and epidemiologic based network suggesting that this did not have a substantial impact on our findings. We did not identify small deletions and insertions; however, these are difficult to identify accurately. It is possible that the cultures had more heterozygosity, which we did not actively investigate, except on the loci that were polymorphic. SNPs in mobile elements and PE and PPE families were excluded from the analysis. These last two account for 10% of the genomic coding potential. Recently, it have been shown that nsSNPs in these families are 3 to 3.3 times more frequent than in non-PE/PPE genes, therefore it is possible that these isolates have mutations in these genes [26].

In conclusion, WGS was useful in determining the mutations that occurred during microevolution of an *M. tuberculosis* strain during a well-documented outbreak. The SNP data were useful to validate the directionality and sequence of transmission that were suggested by the epidemiologic data. Comparative genomic studies will be needed to determine if the mutations observed have any impact on the ability of the bacteria to transmit and cause disease.

## Supporting Information

Table S1 **Primers used to confirm the seven SNPs observed among the nine** ***M. tuberculosis*** **isolates.**
(DOCX)

Table S2 **GenBank Accession Number for each of the single nucleotide polymorphism (SNP) confirmed in each of the patients.**
(DOCX)
