# Supplementary material for: Use of Whole Genome Sequencing to Determine the Microevolution of Mycobacterium tuberculosis during an Outbreak
Source: PLoS One. 2013 Mar 5;8(3):e58235. doi: 10.1371/journal.pone.0058235 (PMC3589338; doi:10.1371/journal.pone.0058235)
Supplement: Table S1 — Primers used to confirm the seven SNPs observed among the nine M. tuberculosis isolates. (DOCX) [file pone.0058235.s001.docx]

**Table S1. Primers used to confirm the seven SNPs observed among the nine *M. tuberculosis* isolates**

| **SNP** | **Forward (5’-3’)** | **Reverse (5’-3’)** |
| --- | --- | --- |
| SNP1 | CCGGAACCATCGTGTATGAG | CATCGGCCACTTTCTTGAAC |
| SNP3 | TCTCGATGTAGCTGTATTCCTTGG | GTCCAAACCCTACCGCAATT |
| SNP4 | TCACGCACAGACCTGCTTTC | GTTGTCGTCACGGACCAATG |
| SNP5 | CGTCCTATTCGTCTGTGTGC | CCAGTACAAATGCGATCCGC |
| SNP6 | AGACTGCGTTCTTGAGCAC | CAGGCATCTTCATGGATTGTCA |
| SNP7 | TGTCTTGCCCACCTTGATCC | CAGCACCAACTGGTAACCATCG |
| SNP8 | TTCGGAGCCCTGACCATTTC | GTAGAGACCGTAGTAACCCACGTA |
