# Supplementary material for: Use of Whole Genome Sequencing to Determine the Microevolution of Mycobacterium tuberculosis during an Outbreak
Source: PLoS One. 2013 Mar 5;8(3):e58235. doi: 10.1371/journal.pone.0058235 (PMC3589338; doi:10.1371/journal.pone.0058235)
Supplement: Table S2 — GenBank Accession Number for each of the single nucleotide polymorphism (SNP) confirmed in each of the patients. (DOCX) [file pone.0058235.s002.docx]

**Table S2. GenBank Accession Number for each of the single nucleotide polymorphism (SNP) confirmed in each of the patients.**

| Sequence identification (SNP number_ patient identification) | GenBank Accession Number |
| --- | --- |
| SNP1_A1 | BankIt1577941 SNP1_A1 KC112849 |
| SNP1_A2 | BankIt1577941 SNP1_A2 KC112850 |
| SNP1_A4 | BankIt1577941 SNP1_A4 KC112851 |
| SNP1_A5 | BankIt1577941 SNP1_A5 KC112852 |
| SNP1_B1 | BankIt1577941 SNP1_B1 KC112853 |
| SNP1_B2 | BankIt1577941 SNP1_B2 KC112854 |
| SNP1_C | BankIt1577941 SNP1_C KC112855 |
| SNP1_D | BankIt1577941 SNP1_D KC112856 |
| SNP1_E | BankIt1577941 SNP1_E KC112857 |
| SNP3_A1 | BankIt1577941 SNP3_A1 KC112858 |
| SNP3_A2 | BankIt1577941 SNP3_A2 KC112859 |
| SNP3_A4 | BankIt1577941 SNP3_A4 KC112860 |
| SNP3_A5 | BankIt1577941 SNP3_A5 KC112861 |
| SNP3_B1 | BankIt1577941 SNP3_B1 KC112862 |
| SNP3_B2 | BankIt1577941 SNP3_B2 KC112863 |
| SNP3_C | BankIt1577941 SNP3_C KC112864 |
| SNP3_D | BankIt1577941 SNP3_D KC112865 |
| SNP3_E | BankIt1577941 SNP3_E KC112866 |
| SNP4_A1 | BankIt1577941 SNP4_A1 KC112867 |
| SNP4_A2 | BankIt1577941 SNP4_A2 KC112868 |
| SNP4_A4 | BankIt1577941 SNP4_A4 KC112869 |
| SNP4_A5 | BankIt1577941 SNP4_A5 KC112870 |
| SNP4_B1 | BankIt1577941 SNP4_B1 KC112871 |
| SNP4_B2 | BankIt1577941 SNP4_B2 KC112872 |
| SNP4_C | BankIt1577941 SNP4_C KC112873 |
| SNP4_D | BankIt1577941 SNP4_D KC112874 |
| SNP4_E | BankIt1577941 SNP4_E KC112875 |
| SNP5_A1 | BankIt1577941 SNP5_A1 KC112876 |
| SNP5_A2 | BankIt1577941 SNP5_A2 KC112877 |
| SNP5_A4 | BankIt1577941 SNP5_A4 KC112878 |
| SNP5_A5 | BankIt1577941 SNP5_A5 KC112879 |
| SNP5_B1 | BankIt1577941 SNP5_B1 KC112880 |
| SNP5_B2 | BankIt1577941 SNP5_B2 KC112881 |
| SNP5_C | BankIt1577941 SNP5_C KC112882 |
| SNP5_D | BankIt1577941 SNP5_D KC112883 |
| SNP5_E | BankIt1577941 SNP5_E KC112884 |
| SNP6_A1 | BankIt1577941 SNP6_A1 KC112885 |
| SNP6_A2 | BankIt1577941 SNP6_A2 KC112886 |
| SNP6_A4 | BankIt1577941 SNP6_A4 KC112887 |
| SNP6_A5 | BankIt1577941 SNP6_A5 KC112888 |
| SNP6_B1 | BankIt1577941 SNP6_B1 KC112889 |
| SNP6_B2 | BankIt1577941 SNP6_B2 KC112890 |
| SNP6_C | BankIt1577941 SNP6_C KC112891 |
| SNP6_D | BankIt1577941 SNP6_D KC112892 |
| SNP6_E | BankIt1577941 SNP6_E KC112893 |
| SNP7_A1 | BankIt1577941 SNP7_A1 KC112894 |
| SNP7_A2 | BankIt1577941 SNP7_A2 KC112895 |
| SNP7_A4 | BankIt1577941 SNP7_A4 KC112896 |
| SNP7_A5 | BankIt1577941 SNP7_A5 KC112897 |
| SNP7_B1 | BankIt1577941 SNP7_B1 KC112898 |
| SNP7_B2 | BankIt1577941 SNP7_B2 KC112899 |
| SNP7_C | BankIt1577941 SNP7_C KC112900 |
| SNP7_D | BankIt1577941 SNP7_D KC112901 |
| SNP7_E | BankIt1577941 SNP7_E KC112902 |
| SNP8_A1 | BankIt1577941 SNP8_A1 KC112903 |
| SNP8_A2 | BankIt1577941 SNP8_A2 KC112904 |
| SNP8_A4 | BankIt1577941 SNP8_A4 KC112905 |
| SNP8_A5 | BankIt1577941 SNP8_A5 KC112906 |
| SNP8_B1 | BankIt1577941 SNP8_B1 KC112907 |
| SNP8_B2 | BankIt1577941 SNP8_B2 KC112908 |
| SNP8_C | BankIt1577941 SNP8_C KC112909 |
| SNP8_D | BankIt1577941 SNP8_D KC112910 |
| SNP8_E | BankIt1577941 SNP8_E KC112911 |
